# A labelling system improves parental comfort and willingness to use topical corticosteroids for paediatric atopic dermatitis

**DOI:** 10.1002/ski2.11

**Published:** 2020-12-29

**Authors:** F. Wilson, E. Harnik, C. Gore

**Affiliations:** ^1^ Department of Medicine Faculty of Medicine Imperial College London London UK; ^2^ Department of Paediatrics Allergies Imperial College Healthcare NHS Trust London UK; ^3^ Section of Inflammation, Repair and Development National Heart and Lung Institute, Imperial College London London UK

## Abstract

**Objectives:**

In the United Kingdom, atopic dermatitis (AD) affects 20% of children and topical corticosteroids (TCS) are a mainstay of AD treatment regimes. Many TCS have similar packaging despite significant differences in potency frequently leading to confusion, and along with misinformation and steroid phobia, potentially reducing treatment adherence. We aimed to evaluate parents' knowledge/concerns regarding TCS and explore benefits of/preference for a TCS‐labelling system.

**Method:**

Hundred parents of children with AD attending paediatric dermatology and/or allergy appointments completed mixed‐methodology Survey 1 (knowledge‐quiz, TCS‐labelling options, feedback on what supports AD‐care). Thirty parents, adolescents, and healthcare professionals completed Survey 2. Qualitative/quantitative data was thematically/statistically analysed (SPSS v25) respectively.

**Results:**

Parents preferred the traffic light system (green = mild, yellow = moderate, red = potent; *n* = 71/100) and reported significantly increased willingness and comfort in using TCS if a labelling system was used *p* ≤ 0.001). Knowledge regarding TCS potency was lacking: 62% (*n* = 46/74) of mild TCS‐users overestimated potency; 51% (*n* = 67/131) of potent TCS‐users underestimated potency. Common concerns were TCS‐related skin thinning, long‐term side effects and themes for improved AD‐care/support included: better information, written plans, access to advice, involvement of certain staff. Parents wanted accessible information in various formats: verbally, electronic resources, leaflets, and education sessions.

**Conclusions:**

Parents of children with AD confirmed significant concerns and demonstrated poor knowledge regarding TCS use. Our findings suggest that a simple labelling system may improve TCS adherence. Future work to test refined label prototypes and evaluating their impact on adherence and correct use is needed.

## 1 BACKGROUND

Atopic dermatitis (AD), is a common chronic relapsing pruritic inflammatory skin condition affecting 1 in 5 children under the age of 10 years in the United Kingdom.^1^, ^2^ Prevalence is rising and it is believed changes in lifestyle and environmental factors could have some influence.^1^, ^2^, ^3^

**What is already known about this topic?**

- AD is a common pruritic skin condition with a treatment regimen which can be confusing for families, and one that many don't adhere to.
- Steroid phobia and confusing steroid packaging compounds poor treatment adherence.

**What does this study add?**

- Quantifies parents' limited understanding of topical steroid potency and safe topical steroid use.
- Evidence that implementation of labelling system could improve treatment adherence.
- A set of recommendations for improvements to paediatric AD services, improving the care that children and families receive, that could be applicable to other centres.

**What are the clinical implications of this work?**

- Highlights the unease of parents and the confusion surrounding safe TCS use for maintenance of AD and management of exacerbations.
- Emphasises the need for solutions to improve misinformation, hesitancy and steroid phobia.
  This includes clear labelling of potency on steroid packaging.
- Parents want detailed, accessible information on AD in various formats: verbally, labelling systems, electronic resources, leaflets, and education sessions.

AD has the greatest impact on quality of life (QoL) compared to other chronic paediatric dermatological conditions.^4^ It is a major source of discomfort for patients and families, mainly due to the impact on activities of daily living, and quality of sleep.^5^, ^6^ There is an increased risk of developing mental health conditions, behavioural problems and poor school performance.^6^, ^7^ The cumulative impact on parents' QoL and mental health is also significant, with mental health scores of parents with children with AD worse than those of parents with children with psychiatric disorders and equivalent to those of parents with children with severe developmental disability and physical morbidity.^8^, ^9^, ^10^ The impact on QoL emphasises the importance of effective early management.

Like many chronic conditions, AD relies mostly on self‐management, thus making poor adherence one of the main identifiable causes of treatment failure.^2^ The routine of applying emollients and topical corticosteroids (TCS) can be onerous, messy, and confusing for families.^11^ One study found the average adherence of an 8‐week treatment regime (involving emollients and TCS) was only 32%.^12^ Although predominantly managed in primary care, patients with severe or refractory disease may require management input from secondary care services such as our study setting, a paediatric allergy and dermatology service.

Poor adherence in AD is multifactorial. A recent UK qualitative study suggested several contributing factors were; confusion about topical application, limited use of written information, conflicting aetiology and management advice from healthcare professionals (HCPs), and lack of self‐management.^13^

Confusion may increase when treatment plans feature multiple TCS with differing potencies depending on disease severity and the intended body application site. This confusion is confounded by the absence of clear indication of TCS potency on packaging. A previously unexplored solution for this is a labelling system, denoting different potencies. A labelling system could provide an inexpensive, simple and quick reminder of the specific steroid potency and its appropriate/intended body site for use, whilst facilitating use in individuals who may not read or speak English fluently. Effective labelling enhances children and adolescents' understanding, for example of nutritional values in food, promoting healthier choices.^14^ Similar educational benefits may be discovered in TCS‐labelling systems, potentially improving clinical outcomes and satisfaction with care.

Despite recommendations from the National Institute for Health and Care Excellence (NICE) that potency should be indicated on all TCS containers, parents rarely report experience with such labelling.^2^ The aim of our project was to therefore explore parental views on the introduction of a labelling system

Our study aims to explore the feasibility and potential benefits of a labelling system to help parents understand appropriate TCS use and improve adherence, with the aim of providing the basis for future interventional studies. As another major barrier to good adherence is steroid phobia, our study also explores parental concerns and understanding of safe TCS use.^15^ Finally, we assess parental understanding of safe TCS use and any suggestions for improvement to their child's AD case.

## 2 METHODS

Parents of children with a clinical diagnosis of AD attending paediatric allergy and dermatology clinics in a secondary/tertiary care setting were invited to participate in an anonymous survey (Survey 1) from January to February 2020. A mixed‐method approach was chosen to allow qualitative and quantitative investigation. Following this, due to COVID‐19 restrictions, a digital anonymous survey (Survey 2) was created and distributed via email to parents, adolescents, and HCPs, to gain feedback on the refined the labelling prototypes.

### 2.1 Survey design

The surveys were devised by Erika Harnik and Claudia Gore, with the assistance of a clinical psychologist to avoid misleading questions. The surveys were piloted with several families and feedback was given. Adjustments were made before formal data collection commenced (See full surveys in Appendix 1 and 2).

### 2.2 Sampling strategy

An initial sample size of *n* = 100 was chosen anticipating that this would allow representation of parental views in the secondary and tertiary care setting and achieve data saturation for qualitative analysis. Parents were chosen as the intended participant as they represent a large proportion of the individuals using TCS on children with AD.

Inclusion criteria:

- Parents or carers of children (under 16 years old) with AD and have used at least one TCS

Exclusion criteria:

- Parents or carers who use TCS for other medical conditions
- Parents or carers of children with AD with no experience of TCS
- Patients with AD over 16 years old

### 2.3 Ethical considerations

All participants were informed the surveys were voluntary, and all data would be reported anonymously. No patient identifiable information was collected. The surveys were interviewer administered by FW, a student researcher, who did not have any involvement with the patient's clinical management. Permission for this service improvement study was granted by the Clinical Governance department of the Trust. Due to the nature of study, consent forms were not necessary.

The study did not meet the criteria requiring NHS Research Ethics Committee review.^16^

### 2.4 Data retrieval

For Survey 1, the objectives of the study were explained, and participants were guided through the survey by FW to ensure queries were addressed and surveys were completed accurately. On completion, surveys were passed to participants to verify and complete the open‐text questions in their own words. For Survey 2, the information was sent via email and participants completed the survey electronically.

### 2.5 Data processing

Survey data were populated into Excel by Florence Wilson. Original paper copies were destroyed once data was inputted to avoid any duplicates.

### 2.6 Data analysis

Data analysis were completed using SPSS v25. Quantitative data were analysed using descriptive statistics:

- If the data were normally distributed, a paired *t*‐test would have been used
- If the data were skewed, a Wilcoxon signed‐rank test would have been used

A *p*‐value of <0.05 was deemed the cut off for rejecting the null hypothesis.

**FIGURE 1:**
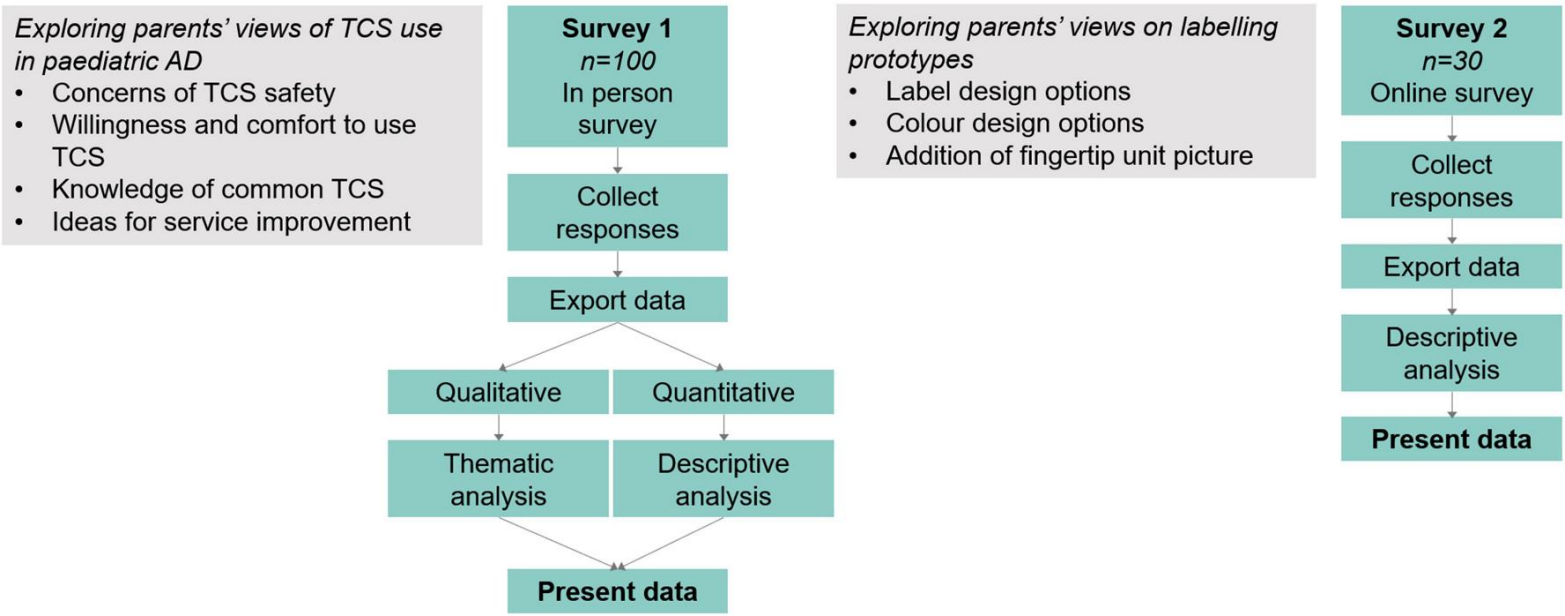
Flow diagram depicting the method for Survey 1 and 2; AD, atopic dermatitis; TCS, topical corticosteroids

Qualitative data from the open‐text questions were coded by FW using NVivo, checked by Erika Harnik, and thematically analysed using Creswell's coding framework (Figure 1).^17^

## 3 RESULTS

Hundred surveys were completed for Survey 1, and 30 completed for Survey 2.

### 3.1 Labelling options

**FIGURE 2:**
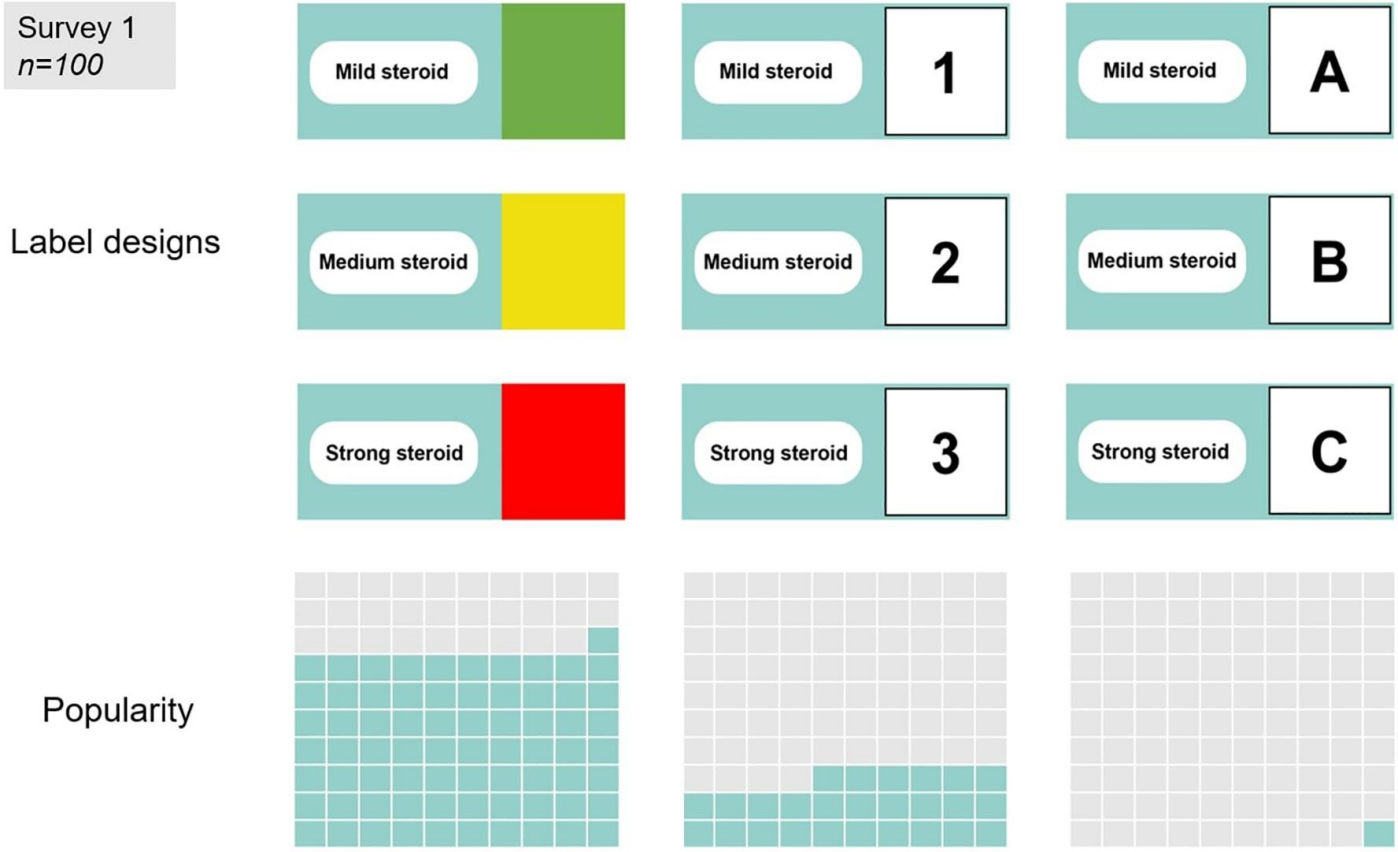
Survey 1—Popularity of the three labelling options surveyed: colours (traffic lights), numerical, alphabetical*; *grey box represents total participants

The most popular labelling system was colours (*n* = 71), 26 preferred numerical, and only 1 participant preferred alphabetical (Figure 2).

**FIGURE 3:**
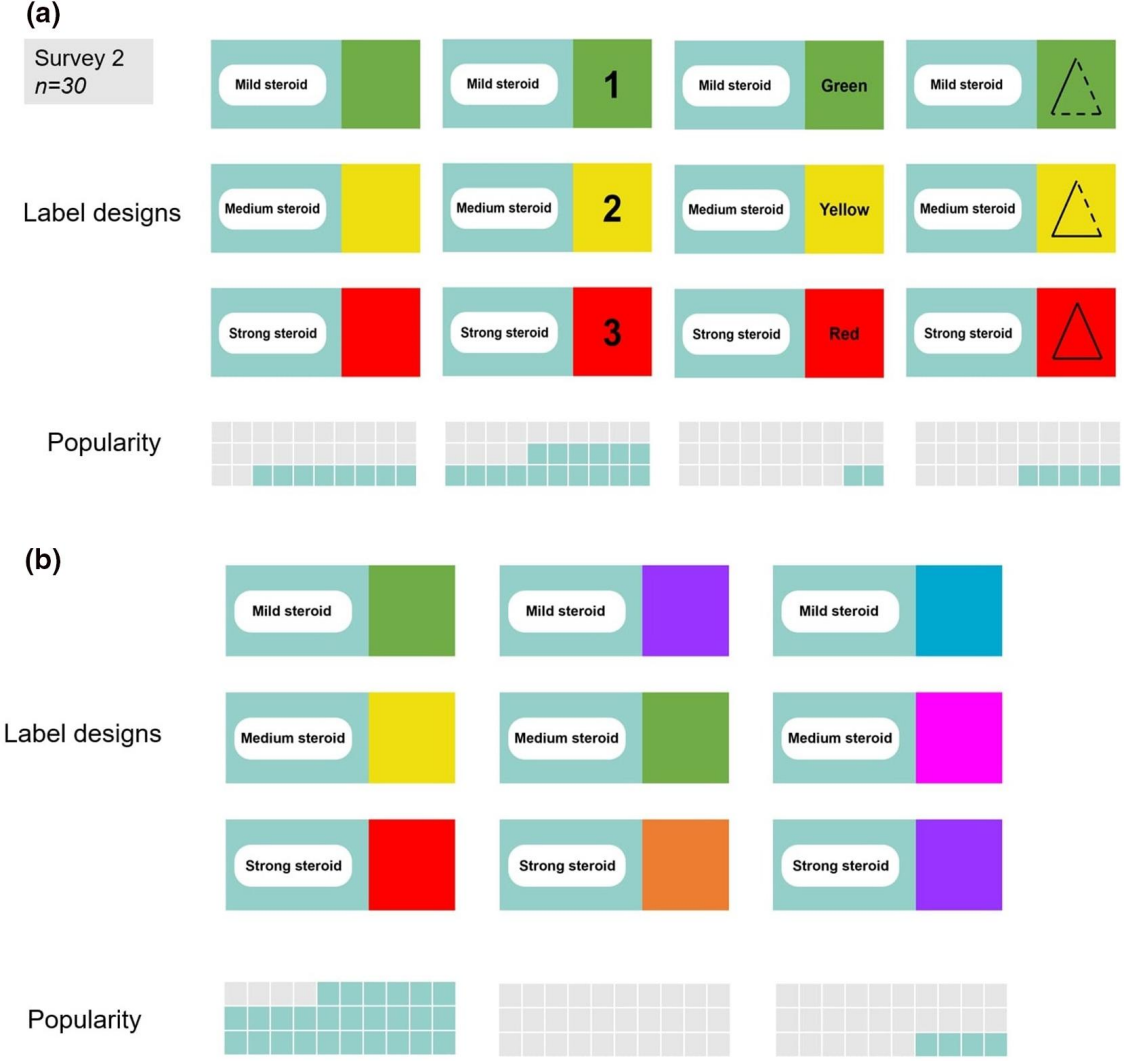
Survey 2—Exploring labelling prototypes. (a) Design options: colours, with numbers, with words, with symbols. (b) Colouring design options: traffic lights, bright colours and cool colours

From Survey 2, the most popular system was the combination of colours and numerical. The most popular colour design was traffic lights (Figure 3).

### 3.2 Views on safe TCS use

Scores of willingness and comfort to use TCS for paediatric AD were recorded from 1 (*very uncomfortable/unwilling*) to 5 (*very comfortable/willing*).

- Twenty said they were ‘*uncomfortable’* to use TCS, none were ‘*very uncomfortable*’
- Eleven said they were ‘*unwilling*’ to use TCS and 2 said they were ‘*very unwilling*’.
- Of the 22 who were ‘*uncomfortable*’, only 6 stated they were ‘*unwilling*’, reflecting the complexity of poor adherence as not all participants who were uncomfortable were unwilling to use TCS if clinically necessary.

As the data was skewed, a Wilcoxon signed‐rank test was calculated and indicated comfort levels ‘after’ the suggestion of a labelling system (median of 5, ‘*very comfortable*’) were higher than comfort levels ‘before’ (median of 4, ‘*comfortable*’) with a statistical significance of *Z* = −7.365 and *p* < 0.001.

Similarly, a Wilcoxon signed‐rank test indicated willingness levels ‘after’ were higher than willingness levels ‘before’ with a statistical significance of *Z* = −7.462 and *p* < 0.001.

**FIGURE 4:**
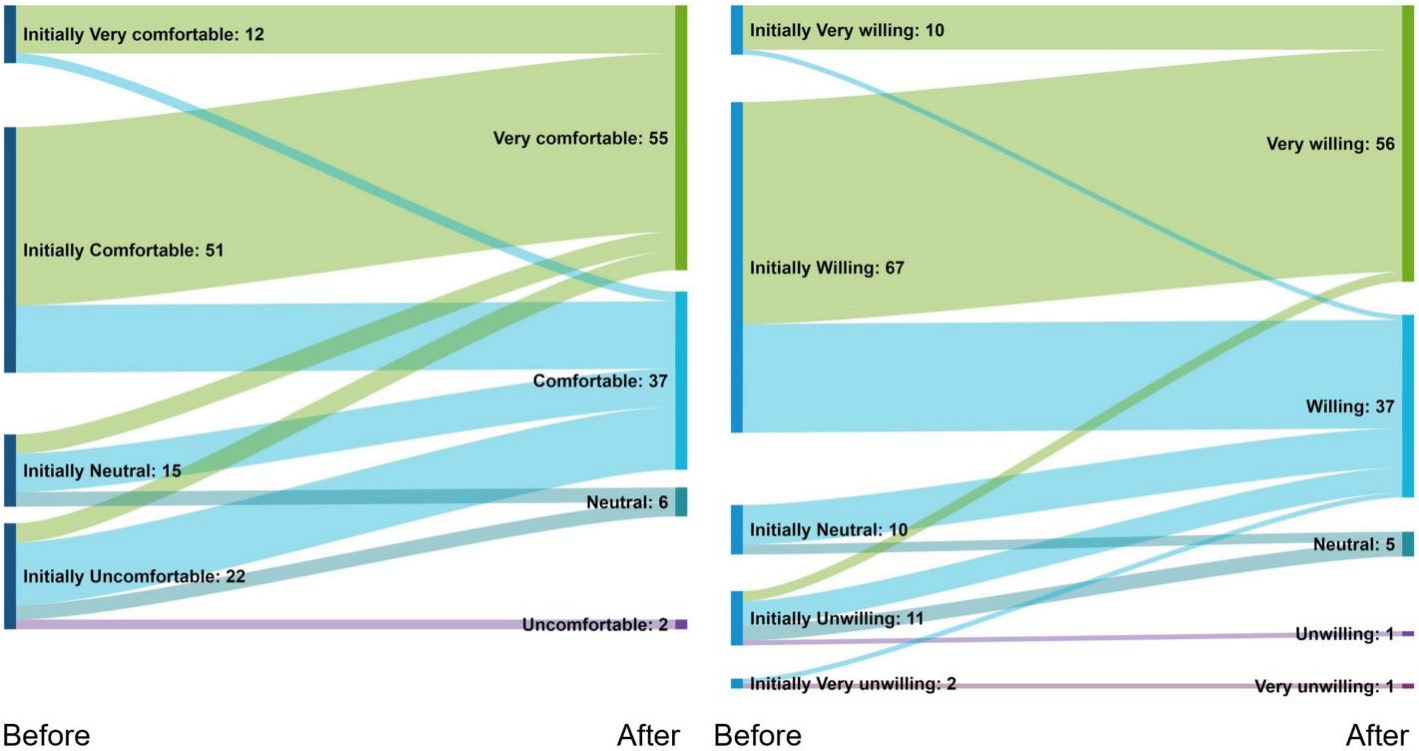
Survey 1—The willingness and comfort scores of participants (*n* = 100) improved ‘after’ the suggestion of a labelling system*; *Sankey diagram shows participants as lines, width is representative of number of participants, and shows the direction of participants’ change in willingness and comfort scores

As there was a statistically significant increase in willingness and comfort scores following the suggestion of a labelling system, the null hypothesis was rejected (Figure 4).

Sixty‐four participants listed one or more TCS concerns. The most common were skin thinning (*n* = 25), general long‐term side effects (*n* = 24), dependence/tolerance (*n* = 10), and skin bleaching (*n* = 7) (data available in Appendix 4 and 5).

### 3.3 Knowledge of safe TCS use

**FIGURE 5:**
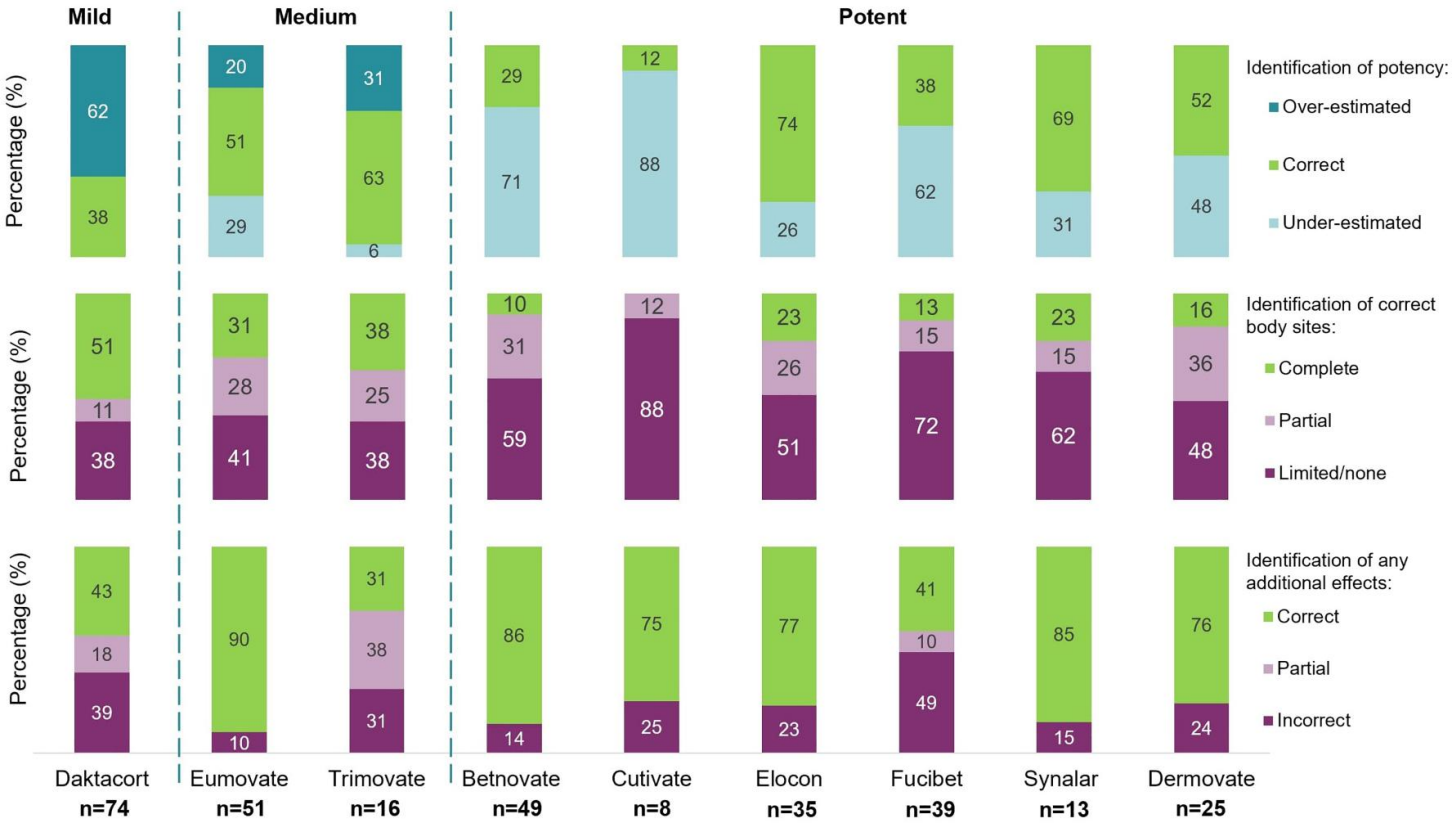
Percentage of participants who correctly identified each topical steroid (TCS) potency, appropriate site on body for use, and any additional antifungal or antibacterial effects* (a) Potency. (b) Correct site of body for use. *Most creams have multiple answers*, *so participants displayed either complete*, *partial or limited knowledge*. (c) Any additional effects. *Some creams have antifungal or antibacterial additional effects therefore the answer can be correct*, *partially correct or not*. *Correct answers can be found in Appendix 3. AD, atopic dermatitis; TCS, topical corticosteroids

The mean number of TCS used was 3.1 (mode: 2, range:1–9.) The most commonly used cream was 1% Hydrocortosone with Miconazole (Daktacort®, *n* = 74). The number of participants who correctly identified a TCS′ potency, additional effects and appropriate site of application varied between creams with some trends identified (Figure 5).

Mild TCS:

- 62% of users overestimated the potency of Daktacort® (*n* = 45).
- The appropriate body sites for use were identified ‘completely’ by 51% (*n* = 38).

Medium TCS:

- The majority who had used 0.05% Clobetasone butyrate (Eumovate®), (51%, *n* = 26) and 0.05% Clobetasone butyrate‐Calcium oxytetracycline and nystatin (Trimovate®), (63%, *n* = 10) correctly identified their potency.
- Only around one third ‘completely’ identified the appropriate body sites for use (31%, *n* = 16 and 38%, *n* = 6 respectively).

Potent TCS:

- The majority who had used 0.1% Betamethasone (Betnovate®), 0.05% Fluticasone propionate (Cutivate®), Mometasone furoate (Elocon®), Fusidic acid‐Betamethasone (Fucibet®), 0.025% Fluocinolone acetonide (Synalar®) and/or 0.05% Clobetasol propionate (Dermovate®) did not correctly identify their potency. Instead, most users underestimated the potency [71% (*n* = 35), 88% (*n* = 7), 26% (*n* = 9), 62% (*n* = 24), 31% (*n* = 4), 48% (*n* = 12), respectively].
- Most users displayed limited or no knowledge of the appropriate body sites for use.

### 3.4 Ideas for improving children's AD care

**FIGURE 6:**
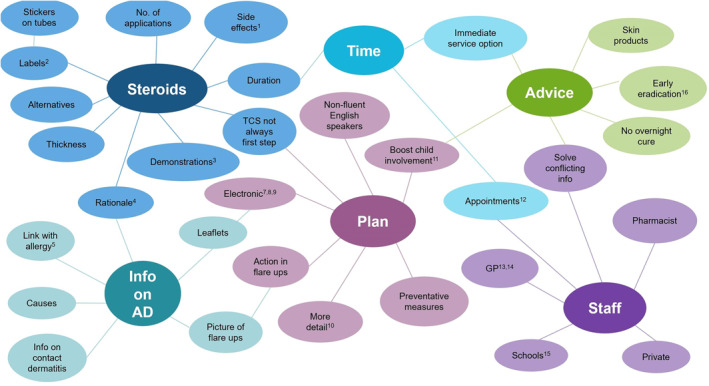
The interlinking themes drawn from the open‐text question inviting participants to suggest ideas for improving their child's AD care and the service they received*. *numbers relate to the example verbatim quotes displayed in Appendix 6; AD, atopic dermatitis

**FIGURE 7:**
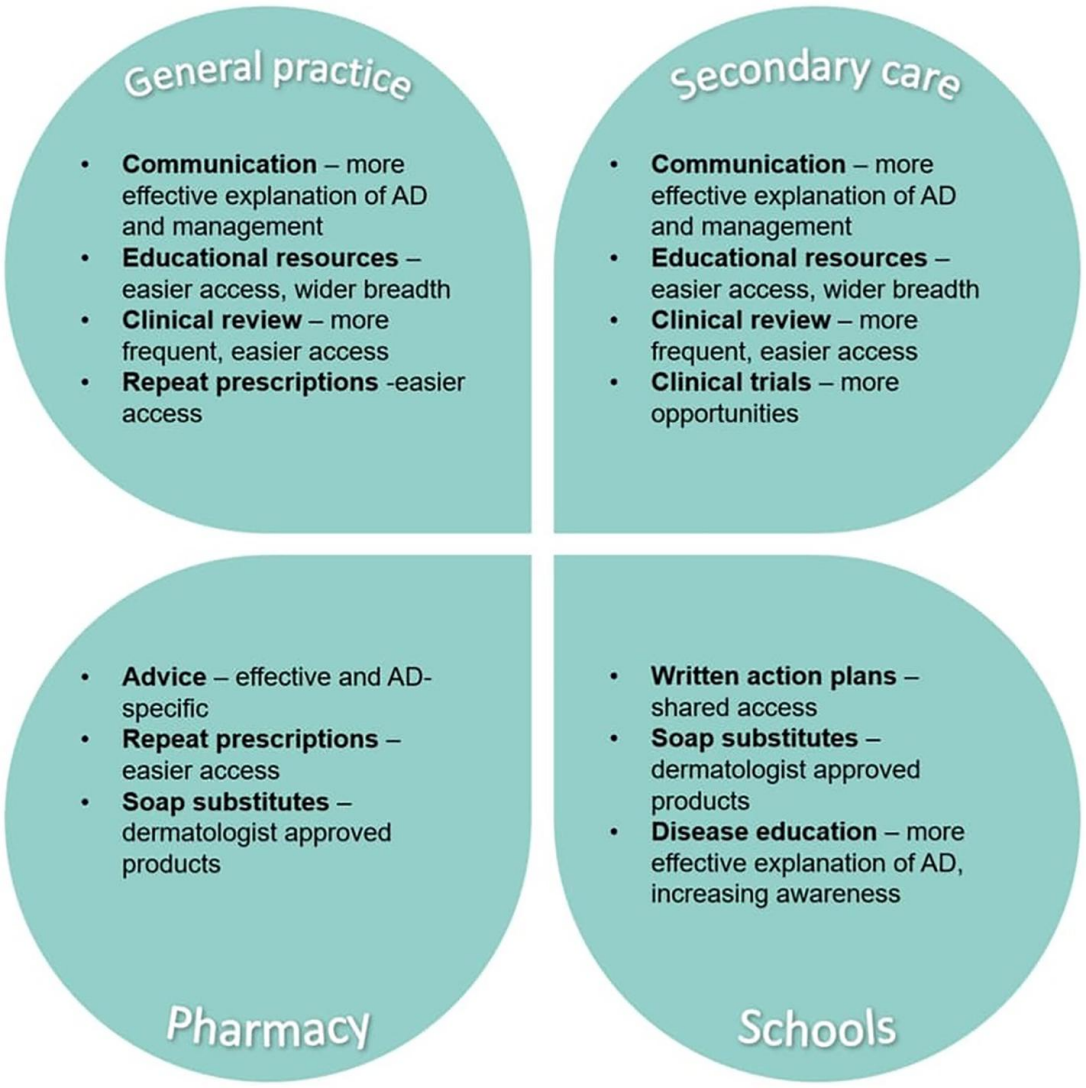
Summary of suggestions for improvement to paediatric atopic dermatitis care and clinical service based on parents' experience in a large inner‐city London hospital secondary and tertiary care paediatric dermatology and allergy service; AD, atopic dermatitis

Sixty‐two participants provided one or more suggestions on how to improve their child's AD care and the service they received. All responses were coded and categorised into one or more theme. The six themes identified were: TCS‐related advice, disease information, time‐related issues, written AD plans, general advice, and improvements specific to certain professional groups (Figure 6). Verbatim quotes were collated to display example suggestions within themes (Appendix 10). A set of recommended improvements specific to our service was created (Figure 7).

## 4 DISCUSSION

Our survey is a novel insight into the potential benefits of a labelling system to help parents feel more willing and comfortable to use TCS for their child's AD. Labels are a quick and simple method to aid recognition of TCS potency, thereby facilitating correct use and limiting the risk of potential adverse effects. Labels may also increase adherence as parents feel reassured their use of TCS is safe, instilling confidence to escalate potency where appropriate. The statistically significant increase in willingness and comfort scores once labels were suggested supports this.

Parents' concerns and lack of knowledge of TCS provides the rationale for the introduction of a labelling system. We found parents underestimated the potency of potent TCS and were unsure where they could be safely applied, potentially increasing the risk of adverse effects. Interestingly, we found parents were also using mild and medium TCS inappropriately, overestimating potency and failing to correctly identify the absence or presence of additional effects. Undertreating AD through TCS hesitancy can lead to prolongation of AD exacerbations and perpetuation of poor clinical outcomes. Hence more must be done to ensure parents understand the safe and effective use of TCS.

Although the most popular colour design was traffic lights, some parents highlighted that ‘red’ for potent steroids had connotations of danger and therefore should be ‘stopped’ like red on a traffic light. However, Survey 2 confirmed parents still endorsed the traffic light system, especially when combined with the numerical system as it made the most logical sense. We are not aware of any published literature on TCS‐labelling systems therefore our original findings will help inform larger, interventional research with the prospect of developing a nationally adopted labelling system.

Our study identifies the essence of parents' concerns and misconceptions regarding TCS use in children, some of which have been previously reported in the literature.^15^, ^18^, ^19^ Fear of steroids is understandable: parents often receive conflicting advice from HCPs, leading to uncertainty, continuation of phobias and poor adherence.^20^ With most participants providing at least one suggestion on how to improve their child's AD care and the service they had received, out study highlights the need for local service

Our service at this inner‐city London hospital, providing secondary and tertiary level allergy and dermatology care, benefits from an expert multidisciplinary team with years of experience. Nevertheless, our study found parents asked for numerous simple improvements thus inferring our service may not be communicating the basics effectively enough. The improvements and information needs have been previously identified in previous studies, but evidently more needs to be done to execute them.^21^ Potential barriers for excellence could include lack of time, funding and availability of experienced staff to run dedicated educational sessions or create informational resources for families. Labelling could be a cost‐effective and time saving tool to improve AD care.

### 4.1 Strengths and limitations

Our project explored a novel labelling system which is acceptable to parents, and the statistically significant increase in willingness and comfort scores confirms it could improve adherence. However, as our project was not a controlled trial, an interventional study is needed to confirm any improved adherence with labels compared to without.

Our survey population consisted of a unique sample of families requiring specialist dermatology and/or allergy care input due to their complexity: severe or refractory disease, or comorbidities. Subsequently, our study may have been subject to selection bias as these families may have more significant adherence issues—due to more complex treatment regimens, more extensive steroid phobia or poor understanding of AD—which will be reflected in the willingness and comfort scores. Our significant findings will help inform further research in the wider AD population, including families receiving treatment wholly in primary care.

In Survey 1, we exclusively surveyed parents and therefore need to also examine the patients' views and experiences with TCS. Involving children and adolescents in the development of a labelling system will help boost patient involvement, and potentially enhance understanding and adherence. Consequently, Survey 2 was also sent to older paediatric patients. The COVID‐19 pandemic impacted the number of participants completing Survey 2, as >99% of clinic appointments were conducted remotely. Survey 2 represents pilot feedback to inform the development of further formal evaluation.

The improvement in parental willingness and comfort to use TCS after the suggestion of a labelling system may have been the product of the survey process itself. Parents may have felt more comfortable using TCS from the discussions between interviewer and the parent whilst completing the survey and the labels would not have a specific effect. In future, studies investigating the benefits of labels must standardise the information and interview process to ensure no additional information is given to either group that may affect views on using TCS.

Original paper surveys were destroyed however Excel data is available on request.

### 4.2 Implications for research and practice

This study forms the basis for a wider evaluation of patient and parent/carer views on the labelling system prototypes, including participants in all care settings. The final TCS‐labelling system will then need to be tested for efficacy and impact on adherence and AD control in a prospective interventional study.

A similar qualitative study exploring perceptions and understanding of topical calcineurin inhibitors, (e.g., Pimecrolimus and Tacrolimus) where similar misconceptions and fears exist would be beneficial but was delayed due to the COVID‐19 pandemic.

## 5 CONCLUSION

Parents of children with AD confirmed significant concerns and demonstrated poor knowledge regarding TCS use. Our findings suggest that a simple labelling system may help improve TCS adherence. Future work will test the refined labelling system prototypes and formally assess effectiveness in improving adherence and correct use.

## ACKNOWLEDGEMENTS

This research received no specific grant from any funding agency in the public, commercial, or not‐for‐profit sectors.

## CONFLICT OF INTERESTS

No conflict of interests have been declared.

## Supporting information

Supporting Information
