# Supplementary material for: A labelling system improves parental comfort and willingness to use topical corticosteroids for paediatric atopic dermatitis
Source: Skin Health Dis. 2020 Dec 29;1(1):e11. doi: 10.1002/ski2.11 (PMC9060095; doi:10.1002/ski2.11)

**Appendix 1** – Copy of Survey 1

**Eczema and Steroid Creams – Patients & Parents**

We know that eczema management can be complicated and that some parents worry about using steroid creams or other treatment creams.

We invite you to fill out this questionnaire to help us understand how we can help to make the treatment of eczema a little less complicated and help with any concerns about the treatments.

1. We know that some patients might feel uncomfortable about using steroid creams. How would you say you feel about using steroid creams?

| Very uncomfortable | Uncomfortable | Neither | Comfortable | Very comfortable |
| --- | --- | --- | --- | --- |

1. If you do feel uncomfortable about using steroids, could you briefly describe your concerns?
2. How willing do you feel about using steroid creams?

| Very willing | Willing | Neither | Unwilling | Very unwilling |
| --- | --- | --- | --- | --- |

1. Do you recognise any of the steroid creams below? Can you identify the strength of the steroid, whether they have any additional effects and where on the body you might use them?

|  | **Never used** | **Strength** | **Additional effects?** | **Where can you use this?**  **(Tick all that apply)** |
| --- | --- | --- | --- | --- |
| Betnovate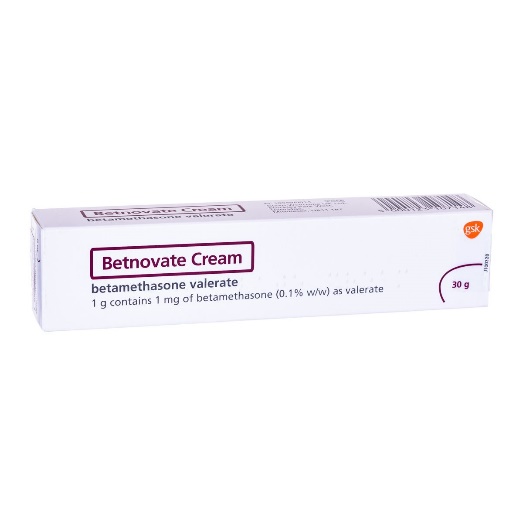 |  | - Mild - Medium - Strong | Anti-bacterial Anti-fungal | Face  Neck  Body  Hands & feet |
| Cutivate  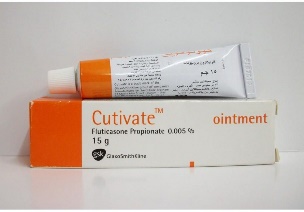 |  | - Mild - Medium - Strong | Anti-bacterial Anti-fungal | Face  Neck  Body  Hands & feet |
| Dermovate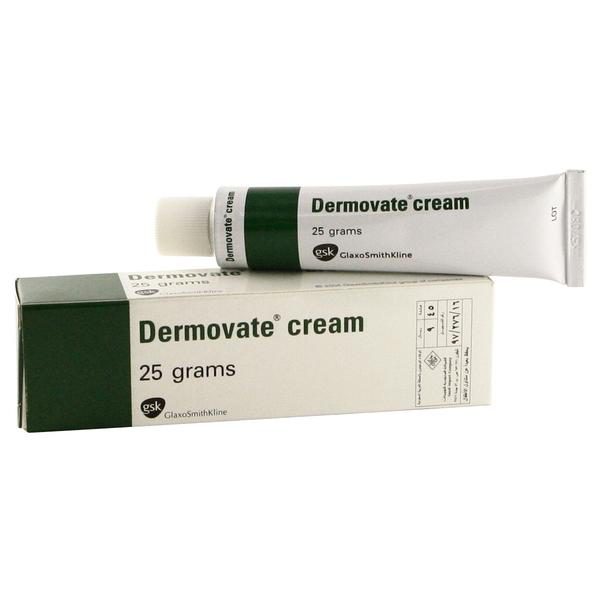 |  | - Mild - Medium - Strong | Anti-bacterial Anti-fungal | Face  Neck  Body  Hands & feet |

| Daktacort 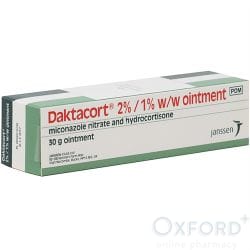 |  | - Mild - Medium - Strong | Anti-bacterial Anti-fungal | Face  Neck  Body  Hands & feet |
| --- | --- | --- | --- | --- |
| Elocon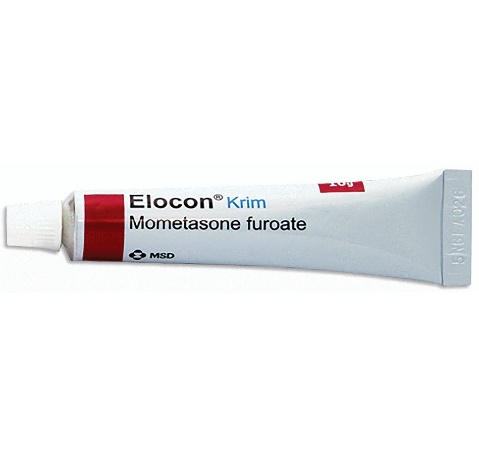 |  | - Mild - Medium - Strong | Anti-bacterial Anti-fungal | Face  Neck  Body  Hands & feet |
| Eumovate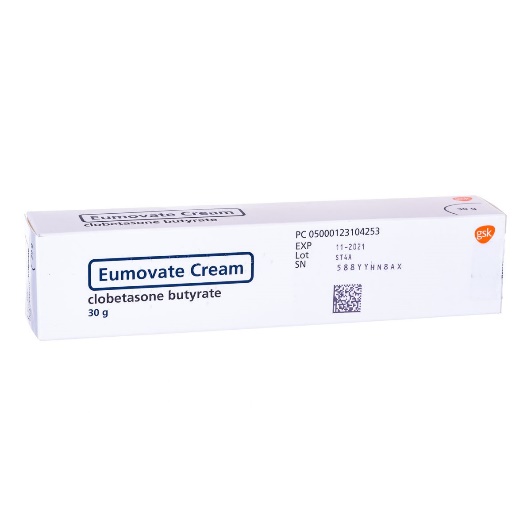 |  | - Mild - Medium - Strong | Anti-bacterial Anti-fungal | Face  Neck  Body  Hands & feet |
| Fucibet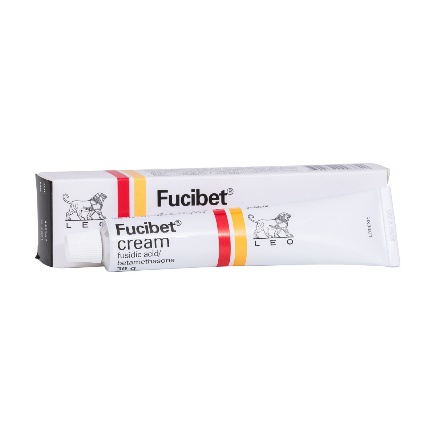 |  | - Mild - Medium - Strong | Anti-bacterial Anti-fungal | Face  Neck  Body  Hands & feet |
| Synalar  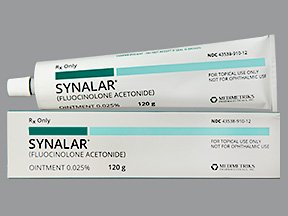 |  | - Mild - Medium - Strong | Anti-bacterial Anti-fungal | Face  Neck  Body  Hands & feet |
| Trimovate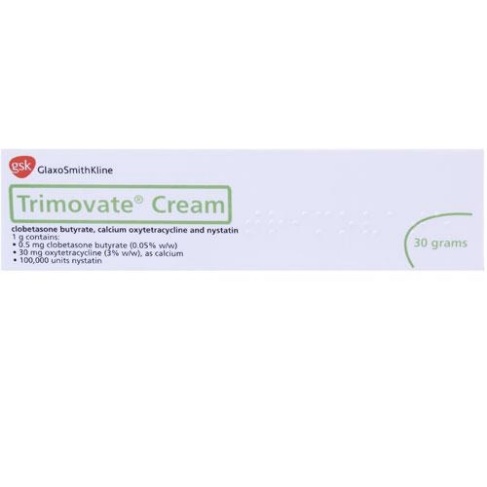 |  | - Mild - Medium - Strong | Anti-bacterial Anti-fungal | Face  Neck  Body  Hands & feet |

1. How do you decide how much steroid to apply?
2. Many patients with eczema have more than one treatment cream. These can be different strengths depending on the severity of the eczema, but the packaging and name does not always help to decide where it can and should be used.

Here are some examples of extra labels to use with steroid creams to help identify different strengths – which ones do you prefer and why?

|  | **Which labelling style do you prefer and why?** |
| --- | --- |
| **OPTION A) Traffic lights (Green / yellow / red)** **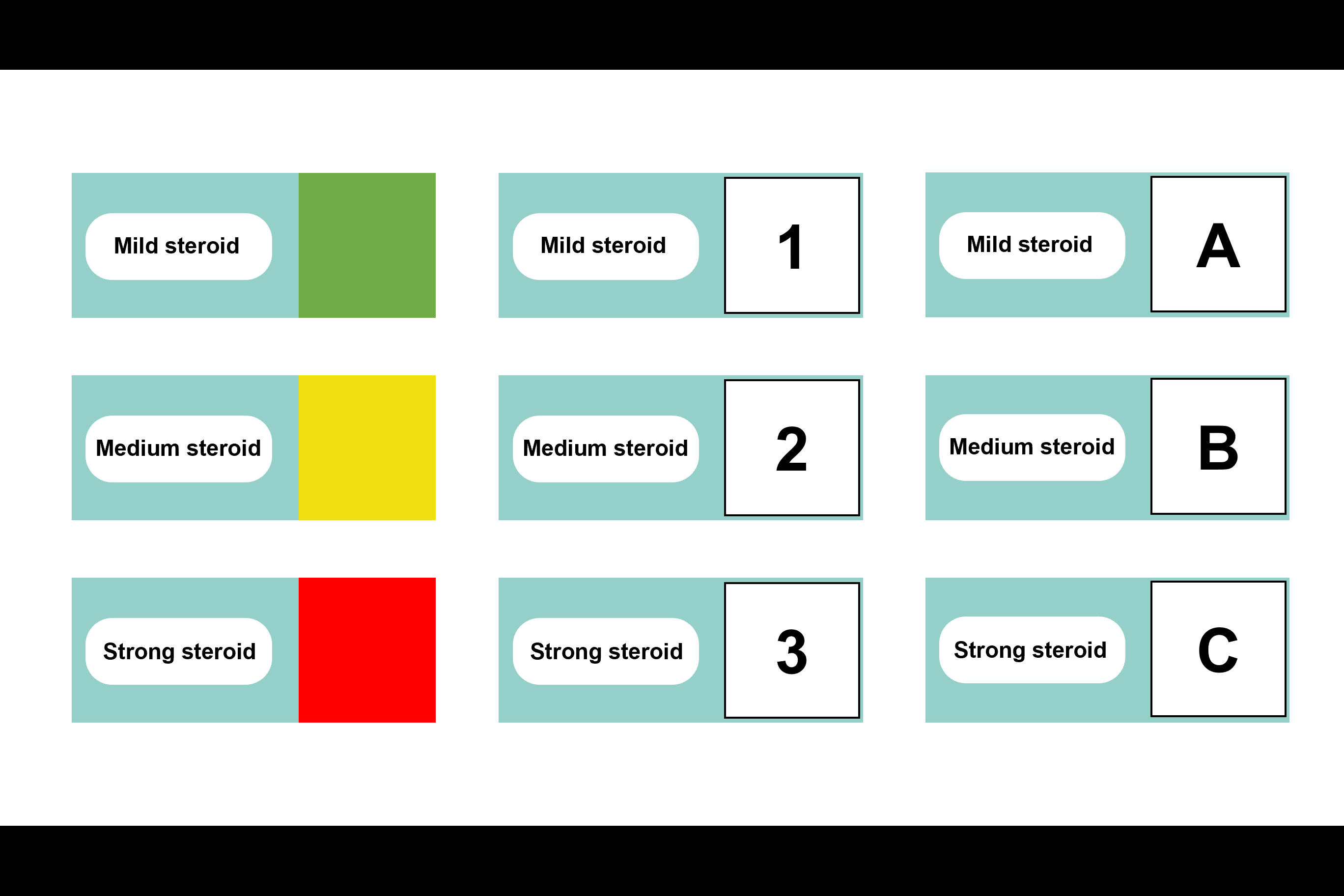** | Please circle your choice:  *Like Neutral Don’t like*  Please tell us why: |
| **OPTION B) Numbers (1,2,3)**  **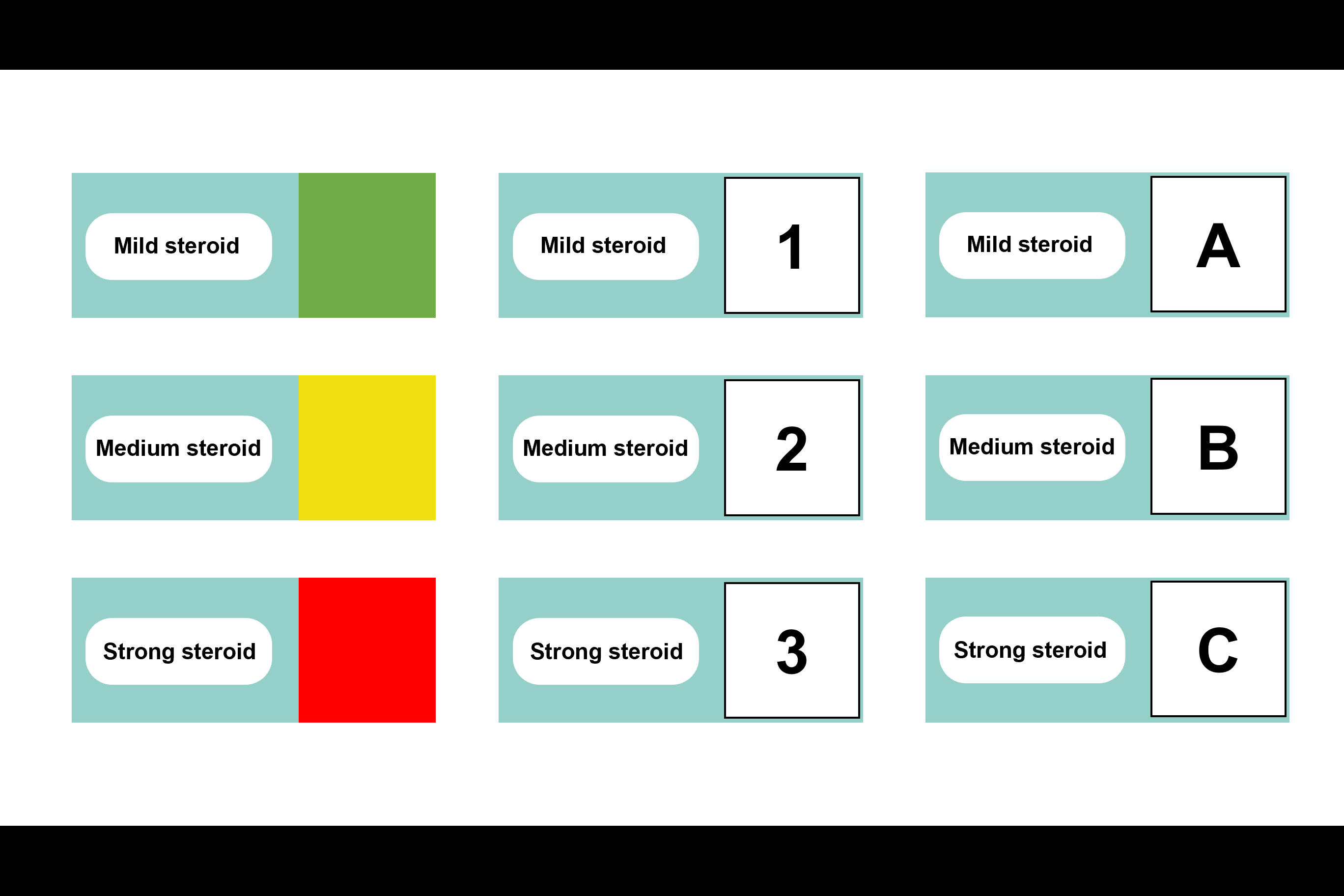** | Please circle your choice:  *Like Neutral Don’t like*  Please tell us why: |
| **OPTION C) Letters (A,B,C)**  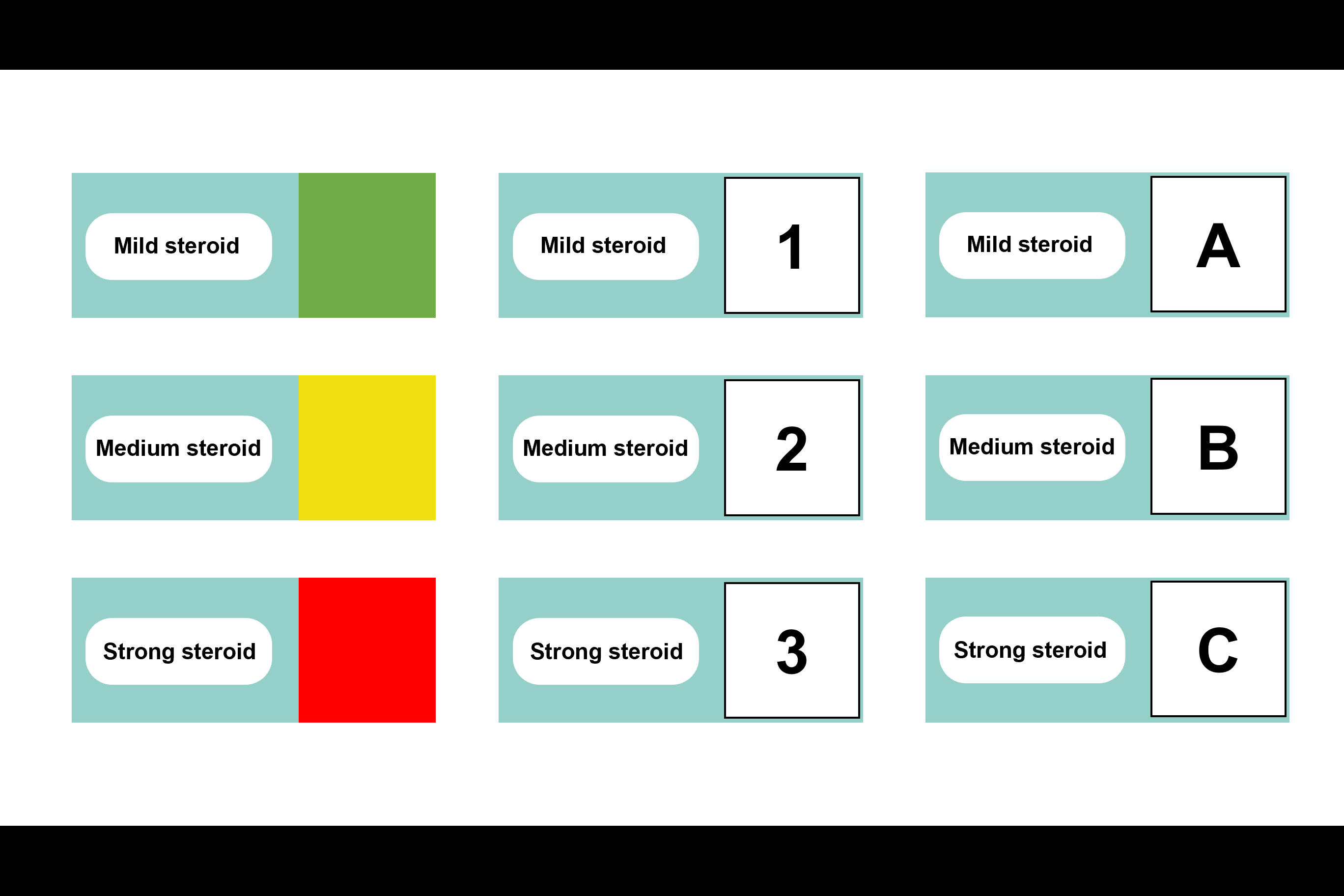 | Please circle your choice:  *Like Neutral Don’t like*  Please tell us why: |

Please rank:

Like best:

Second best:

Like the least:

6B) IF the tubes were labelled as above and you knew the strength how willing would you be to use the creams?

| Very willing | Willing | Neither | Unwilling | Very unwilling |
| --- | --- | --- | --- | --- |

6C) If the tubes were labelled as above, how comfortable would you be using the creams?

| Very uncomfortable | Uncomfortable | Neither | Comfortable | Very comfortable |
| --- | --- | --- | --- | --- |

1. How would you like to learn about steroid creams in eczema (tick any that apply)

- A specific leaflet on steroid creams
- As part of a written eczema treatment plan
- Explained verbally and also give written info
- Other (please specify) …………………………………………………………………………………

1. How well do you feel you understand your child’s eczema management plan?

| I am confident I know what I’m doing | I am somewhat confident | I’m neither confident nor worried | I don’t feel confident | I am really worried and do not feel confident at all |
| --- | --- | --- | --- | --- |

1. How do you feel we could help? Please tell us in your own words:

**Appendix 2** – Copy of Survey 2

**Labelling Options for steroid creams**

We invite you to fill out this questionnaire to help us understand which labelling system would be best for patients and families alike. If you are not currently using steroid creams for eczema (or your child's eczema), we still welcome your thoughts!

- Please state your role
  1. Patient
  2. Parent/carer
  3. Healthcare professional
  4. Other
- If you are a healthcare professional, please specify your position

……………………………………………………………………………………………………………………………………

Labelling systems
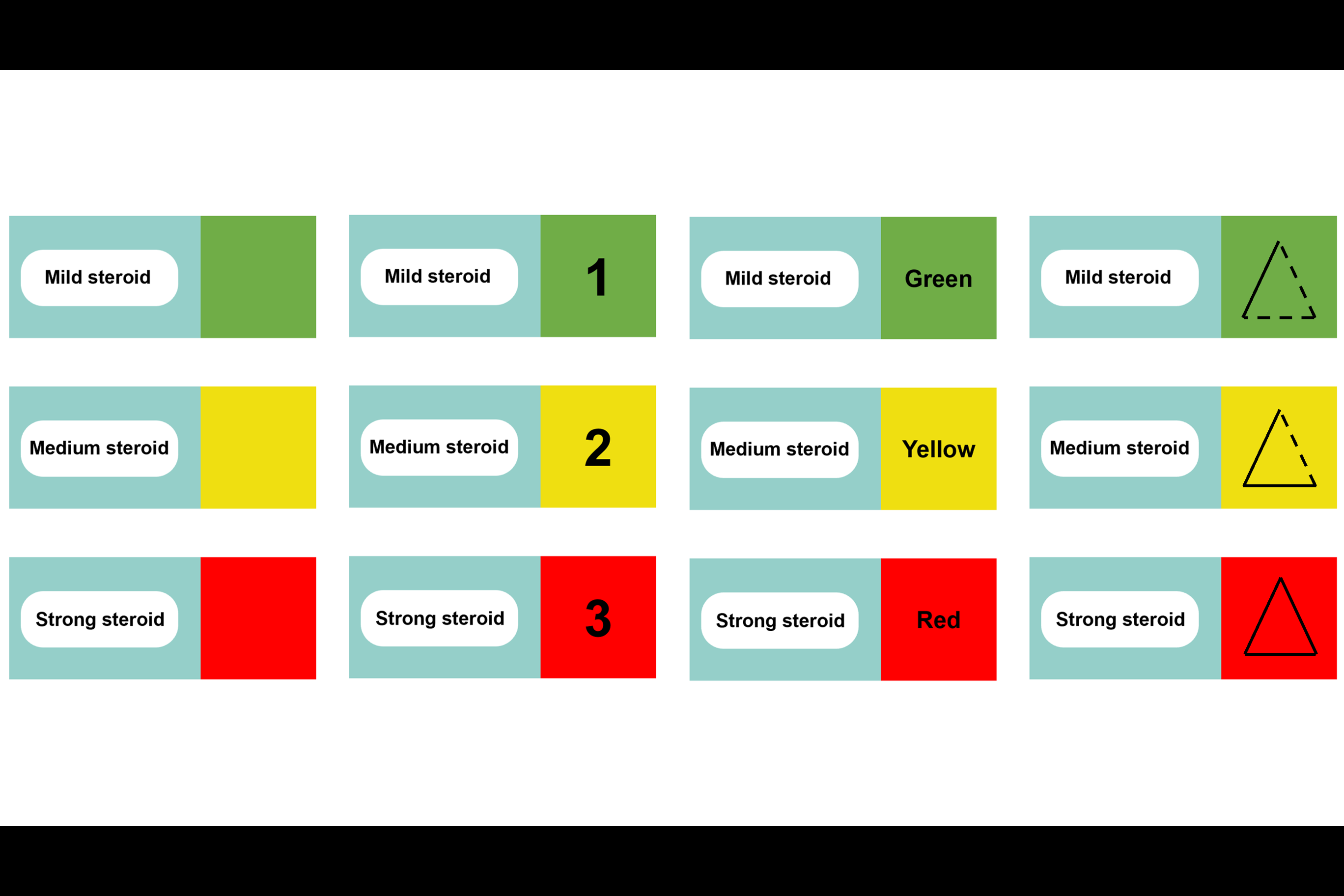


Question 1- Please rank the four labelling systems in order of preference

1^st^ choice:

2^nd^ choice:

3^rd^ choice:

4^th^ choice

PTO

Colour designs


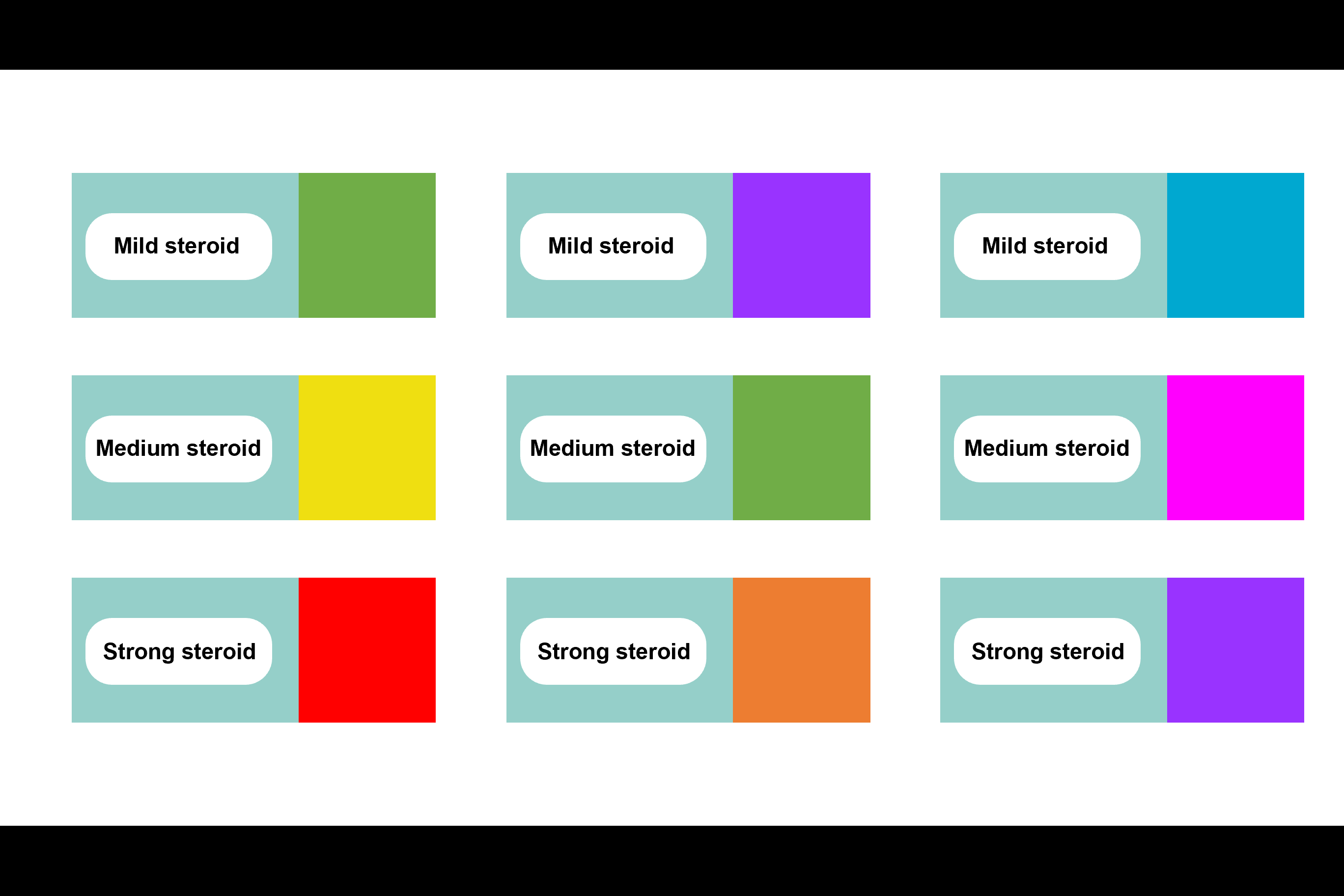


Question 2 - Please rank the three colour design options in order of preference

1^st^ choice:

2^nd^ choice:

3^rd^ choice:

Any comments:

Question 2 - If **none** of these options in question 1 and 2 are satisfactory, ***could you briefly describe why?***

……………………………………………………………………………………………………………………………………………………………………………………

……………………………………………………………………………………………………………………………………………………………………………………

……………………………………………………………………………………………………………………………………………………………………………………

PTO

**Fingertip measurement**


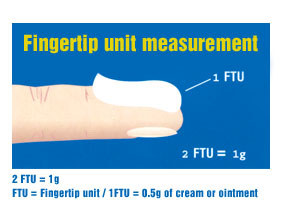


Eczema Clinic, irishhealth.com (<http://www.irishhealth.com/eczema/showdoc.php?m=3&eczid=6344&art_list=P>)

Question 4 – Would you like a picture of the fingertip unit included on the packaging?

| Yes | No |
| --- | --- |

Thank you very much for completing our questionnaire!

**Appendix 3** – Correct answers for identification of different TCS

| **Category** | **Topical Corticosteroid Cream** | **Brand name** | **Potency** | **Any additional effects** | **Correct site of body for use** |
| --- | --- | --- | --- | --- | --- |
| Mild | 1% Hydrocortisone-Miconazole | Daktacort® | Mild | **Anti-fungal** | **Face**, **Neck**, Body, Hands & Feet |
| Medium | 0.05% Clobetasone butyrate | Eumovate® | Medium | No additional effect | **Face**, **Neck**, Body, Hands & Feet |
|  | 0.05% Clobetasone butyrate-Calcium oxytetracycline and nystatin | Trimovate® | Medium | **Anti-fungal** and **anti-bacterial** | **Face**, **Neck**, Body, Hands & Feet |
| Potent | 0.1% Betamethasone valerate | Betnovate® | Potent | No additional effect | Body, Hands & Feet |
|  | 0.05% Fluticasone propionate | Cutivate® | Potent | No additional effect | Body, Hands & Feet |
|  | Mometasone furoate | Elocon® | Potent | No additional effect | Body, Hands & Feet |
|  | Fusidic acid-Betamethasone | Fucibet® | Potent | **Anti-bacterial** | Body, Hands & Feet |
|  | 0.025% Fluocinolone acetonide | Synalar® | Potent | No additional effect | Body, Hands & Feet |
|  | 0.05% Clobetasol propionate | Dermovate® | (very) Potent | No additional effect | Body, Hands & Feet |

**Appendix 4** – Word map* of depicting parents’ concerns regarding the use of TCS on children with AD. *Size of words representative of the number of participants who mentioned each concern

**
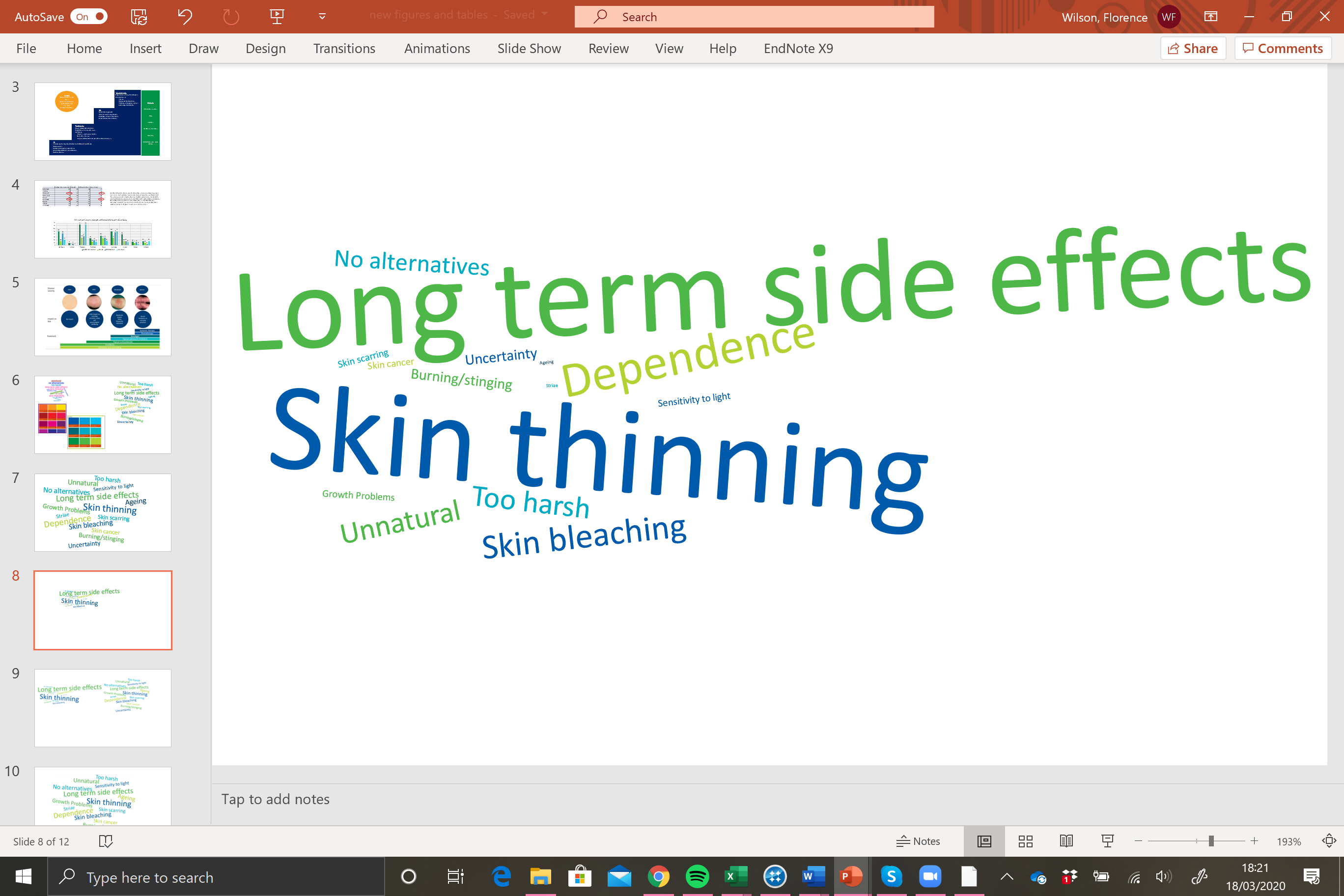
**

**
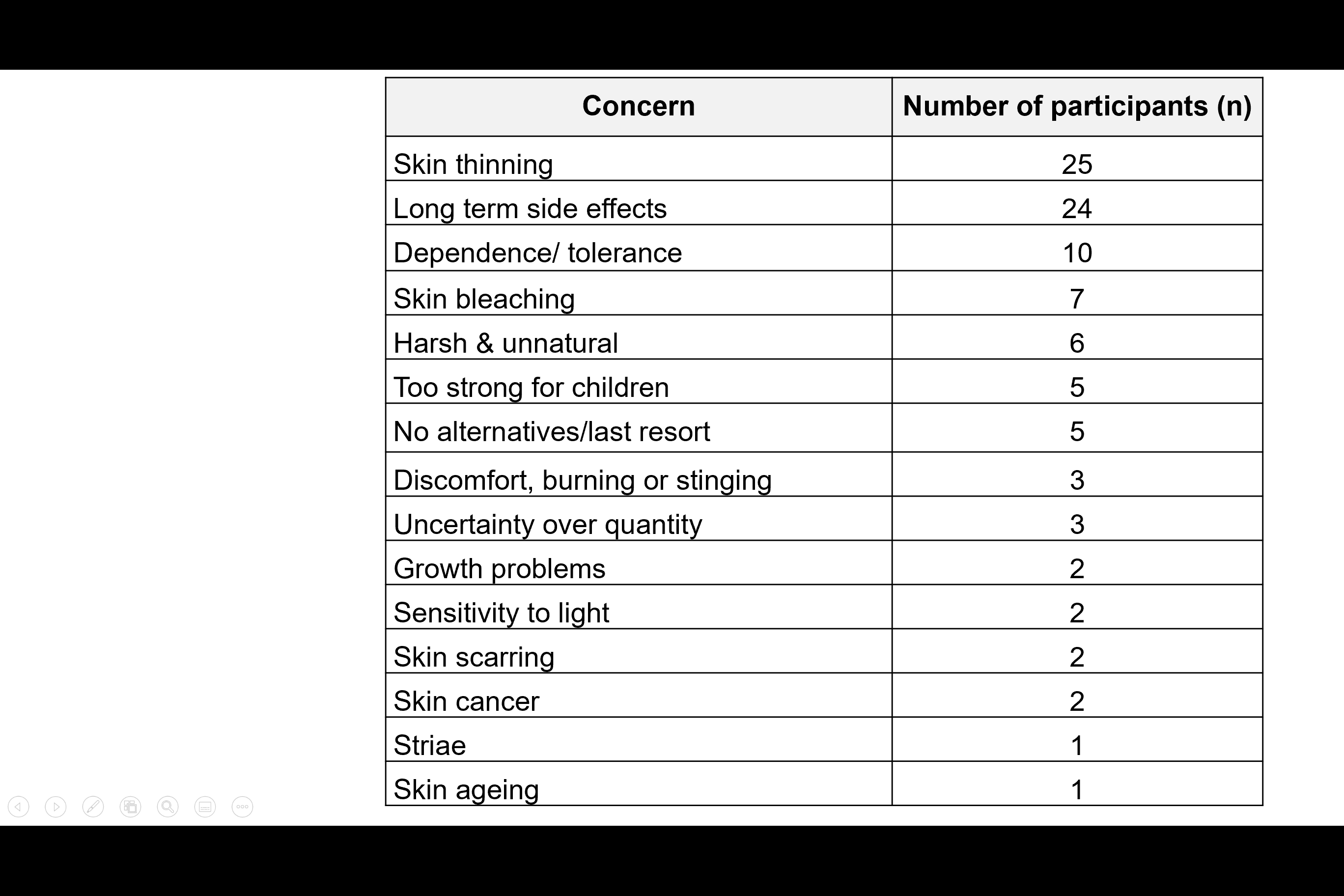
Appendix 5** – List of parents’ concerns regarding the use of TCS on children with AD

**Appendix 6** – Verbatim quotes that reflect the ideas suggested within themes - drawn from the open-text question*.

*Pt = participant


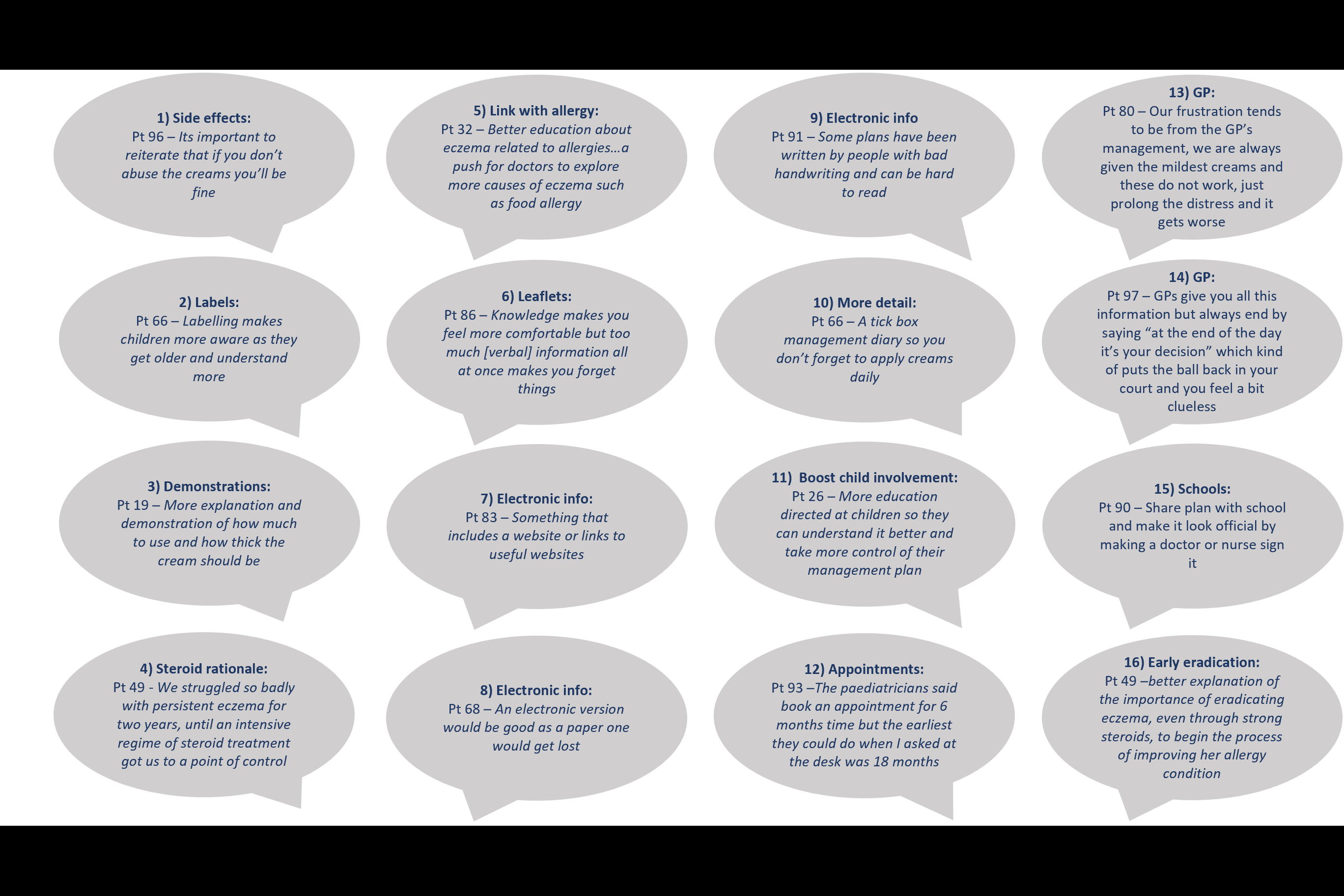

Supplement: Supplementary file 1 — Supporting Information [file SKI2-1-e11-s001.docx]
